# Core Muscle Activity during TRX Suspension Exercises with and without Kinesiology Taping in Adults with Chronic Low Back Pain: Implications for Rehabilitation

**DOI:** 10.1155/2015/910168

**Published:** 2015-06-21

**Authors:** Shirley S. M. Fong, Y. T. Tam, Duncan J. Macfarlane, Shamay S. M. Ng, Young-Hyeon Bae, Eleanor W. Y. Chan, X. Guo

**Affiliations:** ^1^Institute of Human Performance, University of Hong Kong, Pokfulam, Hong Kong; ^2^Department of Rehabilitation Sciences, The Hong Kong Polytechnic University, Hung Hom, Hong Kong; ^3^Department of Physical and Rehabilitation Medicine, Samsung Medical Center, Sungkyunkwan University School of Medicine, Seoul, Republic of Korea; ^4^Department of Physical Therapy, Angelo State University, San Angelo, TX, USA; ^5^Hong Kong Physiotherapists' Union, Central, Hong Kong

## Abstract

This study aimed to examine the effects of kinesiology taping (KT) and different TRX suspension workouts on the amplitude of electromyographic (EMG) activity in the core muscles among people with chronic low back pain (LBP). Each participant (total *n* = 21) was exposed to two KT conditions: no taping and taping, while performing four TRX suspension exercises: (1) hamstring curl, (2) hip abduction in plank, (3) chest press, and (4) 45-degree row. Right transversus abdominis/internal oblique (TrAIO), rectus abdominis (RA), external oblique (EO), and superficial lumbar multifidus (LMF) activity was recorded with surface EMG and expressed as a percentage of the EMG amplitude recorded during a maximal voluntary isometric contraction of the respective muscles. Hip abduction in plank increased TrAIO, RA, and LMF EMG amplitude compared with other TRX positions (*P* < 0.008). Only the hamstring curl was effective in inducing a high EMG amplitude of LMF (*P* < 0.001). No significant difference in EMG magnitude was found between the taping and no taping conditions overall (*P* > 0.05). Hip abduction in plank most effectively activated abdominal muscles, whereas the hamstring curl most effectively activated the paraspinal muscles. Applying KT conferred no immediate benefits in improving the core muscle activation during TRX training in adults with chronic LBP.

## 1. Introduction

Chronic low back pain (LBP) is a common cause of work disability and as such is associated with elevated health care costs [1]. More than 80% of people experience an acute episode of LBP sometime during their lives [2], of which 5% develop chronicity [3]. Core stability training is by far one of the most commonly used rehabilitation strategies for improving lumbopelvic-hip control and the dynamic stability of the lumbar spine in people with chronic LBP [4]. Traditional core stability exercises on a stable surface were found to be less effective in inducing high core muscle activity when compared with core stability exercises performed with an instability device such as the TRX suspension straps [5]. Although the core muscle activity was higher when exercising with the labile suspension straps, the spine compressive load was not high [6, 7]. Therefore, TRX suspension training might be particularly suitable for rehabilitating patients with chronic LBP.

However, among the large variety of TRX suspension workouts [8], which postures are most suitable for individuals with chronic LBP has yet to be determined. There is only one study that has compared the core muscle activity during four different TRX workouts (hamstring curl, hip abduction in plank, chest press, and 45-degree row) in a group of young, healthy individuals (without LBP). The research team found that hip abduction in plank induced the greatest abdominal muscle activity, whereas the hamstring curl induced the greatest paraspinal muscle activity as measured by the surface electromyography (EMG) [9]. In this study, we examined the core muscle activity during the four aforementioned TRX workouts exclusively in individuals with chronic LBP using surface EMG as it can assess muscle activity directly [9]. We hypothesized that different TRX suspension workouts activate the core muscles differentially in people with chronic LBP.

Kinesiology taping (KT) is a relatively new method that has been used in the clinical management of LBP. The tape, which is elastic, porous, and adhesive, can be applied easily to the low back region and does not restrict joint mobility during exercises [10]. Although the effects of KT in clinical studies are controversial [11–14], some beneficial effects of KT have been reported, such as the normalization of muscular function, the correction of possible joint misalignment [11, 12], and enhanced neuromuscular performance [13]. Hence, we postulated that KT might be an ideal adjunct to TRX training to activate the core muscles in people with chronic LBP. To date, no study has examined the acute effects of KT on core muscle activity during the performance of various TRX suspension workouts in individuals with long-term LBP. The aim of this study was to determine the influence of KT and different TRX suspension workouts, and their interactions, on the amplitude of EMG activity in the core muscles among people with chronic LBP.

## 2. Materials and Methods

This was an experimental study during which each participant underwent four TRX suspension workouts/positions (hamstring curl in supine lying with hips lifted, hip abduction in prone plank, chest press, and 45-degree row in standing inclined) and two taping conditions (with and without KT applied to the low back). In each TRX position and taping condition combination, the core muscles' activity was measured using surface EMG. The sequence of TRX workouts was randomized to avoid order effect.

The participants were recruited from the university via convenience sampling. The inclusion criteria were to have chronic LBP, defined as a persisting or periodic pain in the low back region with a duration of six months or longer [15], be between 18 and 30 years old, and have no previous experience in TRX suspension training. The exclusion criteria were to have neurological deficits, spinal structural deformities, genetic spinal disorders, previous spinal surgery, recent spinal or peripheral injuries (e.g., ligament sprains), allergy to KT, or regular consumption of analgesics, or receive active physiotherapy treatments. The study was approved by the Human Research Ethics Committee of the administering university. All of the participants who volunteered to participate in the study were first screened by a physiotherapist and a trained assistant to ensure that the above criteria were met. In addition, all of the experimental procedures and assessment methods were fully explained to each participant before written informed consent was obtained. All of the procedures were performed in accordance with the Declaration of Helsinki.

Demographic information was obtained by interviewing the participants. Body height and weight were measured. Moreover, the participants were asked to quantify their LBP intensity (no pain = 0; worst pain = 10) by using the visual analog scale (VAS) [16]. The self-administered 24-item Roland-Morris Disability Questionnaire (RDQ) was also used to assess physical disability due to LBP (0 = no disability; 24 = maximum disability) [17].

The suspension device, TRX Home Suspension Training Kit (Fitness Anywhere LLC, San Francisco, USA), comprised an adjustable nylon cable with handle straps and foot cradles at both ends that was anchored to the door via a door anchor. The standardized procedures for using the TRX suspension device to perform the four TRX suspension workouts are well described in the TRX user's guide [8]. These four positions were selected for the high activation of core muscles reported in the literature [5, 9]. Body positioning and any adverse effects (e.g., increased in LBP) during the suspension workouts were closely monitored by the research personnel.

Bipolar surface EMG electrodes (EMG sensor SX230-1000, Biometrics, Newport, UK) were used to detect muscle activity among the four major core muscles—transversus abdominis/internal oblique (TrAIO), rectus abdominis (RA), external oblique (EO), and superficial lumbar multifidus (LMF)—on the right side of the body during the TRX suspension exercises. Active electrode placements on the skin were identified following the recommendations of Marshall and Murphy [18] and Barbero et al. [19] and prepared by shaving and cleansing using alcohol swabs to reduce skin impedance. In brief, the positions of the active electrodes were summarized as follows: TrAIO—2 cm inferior and medial to the anterior superior iliac spine; RA—3 cm superior to the umbilicus and 2 cm lateral to the midline; EO—just above the TrAIO electrode site, in direct line with the umbilicus [18]; and LMF—lateral to the midline at the levels of L2 to L4 [19]. The orientation of the active electrodes was parallel to the muscle fibers for optimal signal recording [9]. The reference electrode (R506, Biometrics, Newport, UK) was placed on the ipsilateral tibial tuberosity. Adhesive tapes were applied to ensure all the EMG electrodes were in firm contact with the skin.

The interelectrode distance of the EMG active electrodes was fixed at 1 cm. The EMG signal was sampled at 1000 Hz and amplified by a gain factor of 1000. Other parameters included an input impedance of >10^15^ Ω, common mode rejection ratio of >96 dB, noise of <5 *μ*V, and bandwidth of 20–460 Hz [20]. All of the EMG electrodes and cables were connected to the DataLOG (Biometrics, Newport, UK), which was securely attached to the participant's waist during the TRX workouts to minimize artifacts. The DataLOG used both a high-pass filter (20 Hz) to remove DC offsets due to membrane potential and a low-pass filter for frequencies above 450 Hz. It also stored EMG data for offline analysis [20]. The EMG signals of each core muscle were postprocessed using the Biometrics EMG analysis software for DataLOG version 8.51 (Newport, UK). The root-mean-square value of the EMG signals (EMG_rms_) obtained from each core muscle was calculated.

Before the TRX suspension workouts, EMG data were collected using two five-second maximal voluntary isometric contraction (MVIC) trials against manual resistance for each of the four core muscles with a one-minute, between-trial rest period. Details for applying the manual resistance have been described in Mok et al. [9] and Escamilla et al. [21]. The average EMG_rms_ value during the middle three-second window of each trial was chosen as the representative MVIC value. The MVIC value of each core muscle was recorded for later data normalization [9].

During the TRX-EMG measurement, the participants were required to maintain each of the four TRX positions for five seconds while the EMG activity of the core muscles was being recorded. The RMS of the EMG activity of each core muscle was computed during a middle three-second period during which the participant held his or her position. Two trials were performed for each of the four testing conditions with a one-minute break between trials. The average EMG_rms_ value of two repetitions of each TRX suspension position was normalized against the RMS value of the MVIC of each core muscle, and thus the outcome was expressed as a percentage of MVIC (%MVIC). %MVIC was selected as the major outcome measure because the test-retest reliability was good (ICC = 0.64) [9].

Upon completion of the four TRX suspension workouts and simultaneous EMG recordings, elastic kinesiology taping (k tape, biviax GmbH & Co. KG, Dortmund, Germany) was applied to the low back region. The EMG electrodes were in firm contact with the skin all the time as the KT tapes were applied on top of the adhesive tapes. Each participant received a standardized kinesiology taping application with four I-strips being placed at about 25% tension overlapping in a star shape over the point of maximum pain in the lumbar region [10]. After fixing the tape, all of the above TRX-EMG measurement procedures were repeated. The %MVIC values of the four core muscles (with taping applied) were calculated and used for analysis.

Our statistical analysis was performed using IBM SPSS 20.0 software (IBM, Armonk, NY). The significance level was set at 0.05. A two-way repeated-measures ANOVA (within-subject factors: TRX position and taping condition) was used to compare the normalized EMG_rms_ data (%MVIC) across the different conditions. The Greenhouse-Geisser epsilon adjustment was used if the sphericity assumption was violated. A post hoc paired *t*-test with Bonferroni adjustment was performed if any overall significant results were obtained for the normalized EMG_rms_ data. The effect size (partial eta-squared, denoted as partial *η* ^2^) was also reported—values of 0.14, 0.06, and 0.01 represented large, medium, and small effect sizes, respectively [22].

## 3. Results

**Figure 1:**
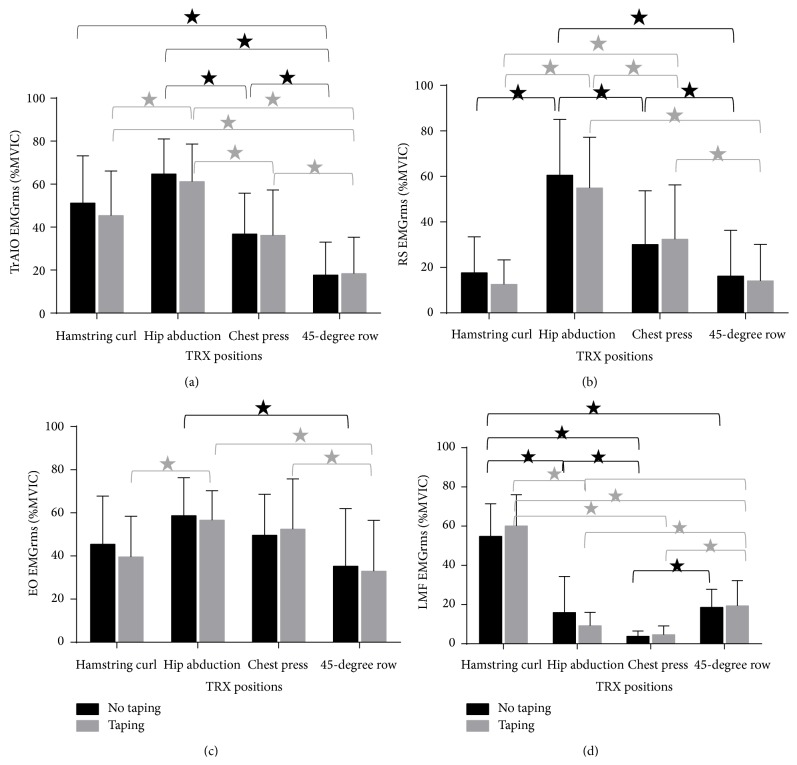
Differences in (a) transversus abdominis/internal oblique (TrAIO), (b) rectus abdominis (RA), (c) external oblique (EO), and (d) lumbar multifidus (LMF) EMG_rms_ amplitudes between different TRX positions and taping conditions. The EMG_rms_ amplitude was expressed as a percentage of that recorded during the MVIC in each test condition (*y*-axis). The values represent the mean and SD. The error bars represent one SD away from the mean. ∗ denotes significant difference (*P* < 0.008, Bonferroni adjusted) between two conditions.

Twenty-one physically active individuals with chronic LBP were screened, and all of them fulfilled the criteria and completed the assessments. No discomfort or adverse events were reported during the suspension workouts. The characteristics of the participants are summarized in Table 1. Regarding the EMG activity of TrAIO, there was an overall significant main effect of the TRX position (*F*_3,60_ = 29.386, *P* < 0.001, partial *η* ^2^ = 0.595), but the main effect of the taping condition (*F*_1,20_ = 1.304, *P* = 0.267, partial *η* ^2^ = 0.061) and the TRX position-taping condition interaction effect (*F*_3,60_ = 0.665, *P* = 0.577, partial *η* ^2^ = 0.032) were not significant. In the post hoc analysis of the main effect of the TRX position, hip abduction in plank induced a significantly higher EMG amplitude than the chest press (*P* < 0.001) and 45-degree row (*P* < 0.001). When taping was added, the hip abduction in plank position induced the highest TrAIO EMG amplitude among all of the tested TRX positions (all *P* < 0.008, Bonferroni adjusted) (Figure 1(a)).

The significant main effect of the TRX position (*F*_2,43_ = 36.243, *P* < 0.001, partial *η* ^2^ = 0.644) was found in the EMG activity of RA. However, the main effect of the taping condition (*F*_1,20_ = 2.119, *P* = 0.161, partial *η* ^2^ = 0.096) and the interaction effect of the TRX position *x* taping condition (*F*_2,45_ = 1.211, *P* = 0.311, partial *η* ^2^ = 0.057) were not significant. Our post hoc analysis of the main effect of the TRX position showed that hip abduction in plank had a significantly higher RA EMG amplitude than the other TRX positions, regardless of taping condition (all *P* < 0.008). Although the chest press induced a higher RA EMG amplitude than the hamstring curl and 45-degree row (regardless of taping condition) (all *P* < 0.008), the resulting RA EMG amplitude was still significantly lower than that obtained during hip abduction in plank (all *P* < 0.008) (Figure 1(b)).

For the EMG activity of EO, the main effect of the TRX position (*F*_2,39_ = 7.495, *P* = 0.002, partial *η* ^2^ = 0.273) was significant. The main effect of the taping condition (*F*_1,20_ = 1.347, *P* = 0.259, partial *η* ^2^ = 0.063) and the TRX position × taping condition interaction effect (*F*_3,60_ = 1.058, *P* = 0.374, partial *η* ^2^ = 0.050) were not significant. A post hoc analysis of the main effect of the TRX position showed that hip abduction in plank elicited a significantly higher EO EMG amplitude than the 45-degree row in the no taping condition (*P* = 0.008). When tape was added, hip abduction in plank induced a significantly higher EO EMG amplitude than the hamstring curl (*P* = 0.006) and 45-degree row (*P* = 0.003). No significant difference in EO EMG amplitude was found between hip abduction in plank and the chest press, regardless of the taping condition (all *P* > 0.008). With taping, the chest press induced a significantly higher EO EMG amplitude than the 45-degree row (*P* = 0.001) (Figure 1(c)).

Regarding the EMG activity of LMF, the TRX position main effect (*F*_3,60_ = 141.422, *P* < 0.001, partial *η* ^2^ = 0.876) and the TRX position × taping condition interaction effect (*F*_2,36_ = 3.785, *P* = 0.036, partial *η* ^2^ = 0.159) were significant. The main effect of the taping condition, however, was not significant (*F*_1,20_ = 0.003, *P* = 0.955, partial *η* ^2^ < 0.001). A post hoc analysis revealed that the hamstring curl resulted in a significantly higher LMF EMG amplitude than all of the other TRX positions in both taping conditions (all *P* < 0.001). Among the other three TRX positions, the 45-degree row resulted in a higher LMF EMG amplitude than the chest press in both the taping (*P* < 0.001) and no taping conditions (*P* < 0.001). The 45-degree row also resulted in a higher LMF EMG amplitude than hip abduction in plank when taping was applied (*P* < 0.001), but this was not the case when the tape was removed (*P* = 0.494). The difference in LMF EMG amplitude was not significant between the two taping conditions across all of the TRX positions (*P* > 0.05) (Figure 1(d)).

## 4. Discussion

This is the first study to investigate the effects of KT and different TRX suspension positions and their interactions on core muscle activity in individuals with chronic LBP. The results reveal that hip abduction in plank induced the highest level of abdominal muscle (TrAIO, RA, and EO) activity (>50% MVIC) in adults with chronic LBP. This finding is primarily in line with a previous study led by Mok et al. [9] who reported that the activity of TrAIO and EO was very high (>60% MVIC) and that of RA was moderately high (close to 40% MVIC) during the hip abduction in plank position in LBP-free participants. The high abdominal muscle activation could be explained by the muscle actions of TrAIO, RA, and EO. In the TRX prone plank position, these muscles contract bilaterally to prevent anterior pelvic tilt and lumbar hyperextension due to the gravitational effect [8, 23].

Interestingly, we found that LMF muscle activation was very low (<20% MVIC) during the hip abduction in plank workout in participants with chronic LBP. This is in contrast to a previous study of back pain-free adults, which showed that LMF activation was moderately high (about 40% MVIC) during hip abduction in plank [9]. The discrepancies in these results may be due to differences in the subject groups. In individuals with chronic LBP, the normal synergistic coactivation pattern of abdominal and paraspinal muscles is lost [24], and wasting [25] and dysfunction of the paraspinal muscles are present [26]. Thus, our participants with chronic LBP demonstrated very low LMF activity during the hip abduction in plank workout. Perhaps this is a TRX training precaution for LBP individuals with weak LMF, as this exercise may cause one to become predisposed to spinal instability and dysfunction [25].

Our results also demonstrate that the hamstring curl is the best choice to elicit high LMF muscle activity (>50% MVIC) in people with chronic LBP. This is in agreement with Mok et al. [9], who reported that LMF activity was the highest (40–60% MVIC) during TRX hamstring curl in LBP-free participants. During the hamstring curl in a supine lying position, lifting the pelvis up (trunk extension) is emphasized [8]. This may increase the activation of the lumbar paraspinal muscles, resulting in a higher LMF EMG amplitude [23].

Similar to the results found in healthy individuals [9], our results showed that, in general, greater core muscle activity was observed in individuals with chronic LBP during the lower limb TRX workouts (hip abduction in plank and hamstring curl) than during the upper limb TRX workouts (chest press and 45-degree row). This finding is logical, because during upper limb workouts the participants stood with a relatively wide and stable base of support and the lower limbs bore most of the body weight [8]; thereby the demand on the core muscles might have been less [23, 27]. Specifically, we found that the chest press was the second best position to induce high muscle activity of EO (the best position was hip abduction in plank), and the 45-degree row was the second best position to elicit high muscle activity of LMF (the best was hamstring curl). Our results hint that the chest press may be an alternative to hip abduction in plank to activate the abdominal muscles and the 45-degree row may be an alternative to the hamstring curl to activate the paraspinal muscle among weaker patients with LBP.

Regarding the immediate effects of KT on core muscle activation during different TRX workouts, the insignificant main effects and interaction effects, together with the small effect sizes, implied that taping may not have been effective in improving core muscle activation during the TRX suspension workouts among individuals with chronic LBP. With a few exceptions, the taping might have enhanced LMF muscle activity during the 45-degree row, EO muscle activity during the chest press, and TrAIO muscle activity during hip abduction in plank. Previous studies have suggested that KT applied longitudinally to the lumbar paraspinal muscles can normalize muscle function through mechanoceptor activation [13]. It is plausible that the taping method employed influenced the EMG results. Further experimental study is definitely required to confirm whether elastic taping (with standardized application procedures) can improve core muscle activation in people with chronic LBP.

The major limitation of this study is that EMG (%MVIC) was used to examine the level of core muscle activation during the TRX exercises. Although a strong EMG-force linearity was reported for the trunk musculature [28, 29], this is only a rough estimation of muscle force production [30, 31]. It is still not clear whether TRX training can actually strengthen the core muscles. Further randomized controlled trials with larger sample size and perhaps longer KT application period are needed to determine the effectiveness of different TRX exercises with and without KT for strengthening the core muscles in adults with chronic LBP. Another limitation of this study is the lack of a parametrized control in the dynamic EMG test. The movement speed during TRX exercises and the participant's effort were hard to be controlled so that the participants might not have delivered their maximal effort during EMG data collection. Therefore, the results should be interpreted with caution.

## 5. Conclusions

The results of this study suggested that TRX hip abduction in plank most effectively activated the TrAIO, RA, and EO, whereas the TRX hamstring curl most effectively activated the LMF in adults with chronic LBP. It seems that applying KT did not have any acute effect on core muscle activation during TRX exercises in this particular group of individuals.

## Figures and Tables

**Table 1:** Characteristics of the participants (*n* = 21).

Variable	Value (mean ± SD)
Basic demographics	
Age, year	21.4 ± 1.7
Sex, men/women, *n*	11/10
Height, m	1.7 ± 0.09
Weight, kg	58.7 ± 11.2
Body mass index, kg m^−2^	20.4 ± 2.3
Low back pain characteristics	
VAS pain intensity score	3.2 ± 1.8
RDQ-24 score	3.4 ± 2.2
MVIC EMG	
TrA/IO, mV	0.20 ± 0.13
RA, mV	0.49 ± 0.25
EO, mV	0.12 ± 0.11
LMF, mV	0.36 ± 0.15
